# Non-Lethal Control of the Cariogenic Potential of an Agent-Based Model for Dental Plaque

**DOI:** 10.1371/journal.pone.0105012

**Published:** 2014-08-21

**Authors:** David A. Head, Phil D. Marsh, Deirdre A. Devine

**Affiliations:** 1 School of Computing, University of Leeds, Leeds, United Kingdom; 2 Microbiology Services, PHE Porton, Salisbury, United Kingdom; 3 Department of Oral Biology, School of Dentistry, University of Leeds, United Kingdom; LSU Health Sciences Center School of Dentistry, United States of America

## Abstract

Dental caries or tooth decay is a prevalent global disease whose causative agent is the oral biofilm known as plaque. According to the ecological plaque hypothesis, this biofilm becomes pathogenic when external challenges drive it towards a state with a high proportion of acid-producing bacteria. Determining which factors control biofilm composition is therefore desirable when developing novel clinical treatments to combat caries, but is also challenging due to the system complexity and the existence of multiple bacterial species performing similar functions. Here we employ agent-based mathematical modelling to simulate a biofilm consisting of two competing, distinct types of bacterial populations, each parameterised by their nutrient uptake and aciduricity, periodically subjected to an acid challenge resulting from the metabolism of dietary carbohydrates. It was found that one population was progressively eliminated from the system to give either a benign or a pathogenic biofilm, with a tipping point between these two fates depending on a multiplicity of factors relating to microbial physiology and biofilm geometry. Parameter sensitivity was quantified by individually varying the model parameters against putative experimental measures, suggesting non-lethal interventions that can favourably modulate biofilm composition. We discuss how the same parameter sensitivity data can be used to guide the design of validation experiments, and argue for the benefits of *in silico* modelling in providing an additional predictive capability upstream from *in vitro* experiments.

## Introduction

Dental caries is a common disease that reduces quality of life globally and represents a substantial economic burden for health organisations [1]. It is caused by acids (primarily lactic) in the oral cavity which initiate demineralisation of tooth enamel, resulting in lesions that can develop into cavities. These acids are produced as a by-product of the glycolysis of dietary sugars by the bacterial community that comprises the oral biofilm known as dental plaque. However, there is no single pathogenic species responsible for caries. Although *Streptococcus mutans* has been widely implicated and studied in this context, carious lesions can exist in the absence of *S. mutans* and be absent in its presence [1], [2]. It is instead thought that any acid-producing species that can metabolise sugars at low pH, *i.e.* those that are both *acidogenic* and *aciduric*, can contribute to the caries process. This description includes *S. mutans*, but also other mutans streptococci, lactobacilli, bifidobacteria, and others. Clinical trials have confirmed the correlation between biofilm composition and caries progression, with healthy and diseased sites associated with distinct subpopulations of bacteria [3]–[5].

The bacterial composition of dental plaque, and biofilms in general, is the result of a dynamic interplay between microbial physiology and external perturbations from the environment and host, and in this sense can be meaningfully regarded as ecosystems [6]–[8]. For oral biofilms, this has been formalised into the *ecological plaque hypothesis* [9]. Varying external conditions therefore alter biofilm composition, potentially increasing or decreasing the fraction of cariogenic species, and *in vitro* studies have shown that the low pH resulting from pulsing glucose into mixed species biofilms causes population shifts favouring *S. mutans* and lactobacilli [10], [11]. This ecological perspective suggests alternative therapeutic strategies: rather than eradication of bacteria, which is both difficult and undesirable as many oral bacteria are also beneficial to the host, it may be possible to instead modulate the biofilm composition to favour bacterial communities with a lower fraction of acidogenic, aciduric species. Indeed, *in vitro* studies have demonstrated that fluoride below lethal concentrations can reduce the fraction of putative cariogenic organisms [12], [13]. The mechanism is thought to be a reduction of their aciduricity and acidogenicity [14]–[17], and reducing their competitiveness with respect to non-pathogenic species such as *S. gordonii* [18]–[20].

Identifying targets to modulate biofilm composition to the benefit of the host is challenging due to the significant complexity of the system, with many coupled mechanisms driving population changes. Systematically varying the many candidate factors in *in vitro* experiments is costly both in time and expense. It is desirable to introduce an additional predictive layer before *in vitro* modelling to highlight promising targets, and this can be realised by *in silico* modelling, *i.e.* computational simulation of mathematical models. Such models generate quantitative predictions over broad ranges of parameter space in relatively short time. In addition, they are not restricted to specific species of bacteria but can systematically incorporate strain and sub-strain variation by continuously varying parameters related to cellular physiology. They are thus well suited to probing populations of species defined by their function, rather than their genetic identity. Early mathematical models of dental plaque by Dibdin *et al.* adopted a continuum approach in which concentrations of various dispersed phases varied smoothly with distance from the enamel surface [21]–[26]. This approach has recently been advanced by Ilie *et al.* who included numerous coupled fields to more realistically represent acid buffering, polyglucose storage *etc.* [27]. These studies did not consider the changes in biofilm composition necessary to study population response to perturbations. Such questions can be naturally addressed using agent based modelling, which is established in biofilm research [28]–[36] but has not yet been applied to plaque.

The aim of this paper is to describe findings from an agent based model of *supragingival* plaque, *i.e.* that component of the oral biofilm above the gumline that is responsible for dental caries, developed to probe the relationship between evolving biofilm composition and cariogenic potential. The model consists of two competing populations of bacteria, one that is pathogenic in that it is both acidogenic and aciduric, and a second non-pathogenic population which, while acidogenic, cannot metabolise sugars at low pH and thus is non-aciduric. These populations are labelled A and NA respectively, for ‘aciduric’ and ‘non-aciduric’. It is found that one population or the other dominates at late times, with a ‘tipping point’ between the two outcomes that depends on a range of parameters relating to both intrinsic physiological processes and external factors such as the frequency of sugar intake. Treatments intended to modify these parameters could therefore drive plaque composition towards a healthy, non-cariogenic state. In addition, sensitivity analysis reveals the relative importance of each model parameter on putative experimental measurements which, as well as highlighting the most important mechanisms relevant to plaque function, can also guide validation experiments by identifying parameter-measurement pairings with high sensitivity, suitable for fixing input parameters from *in vitro* data.

## Results

### Interspecific competition

**Figure 1:**
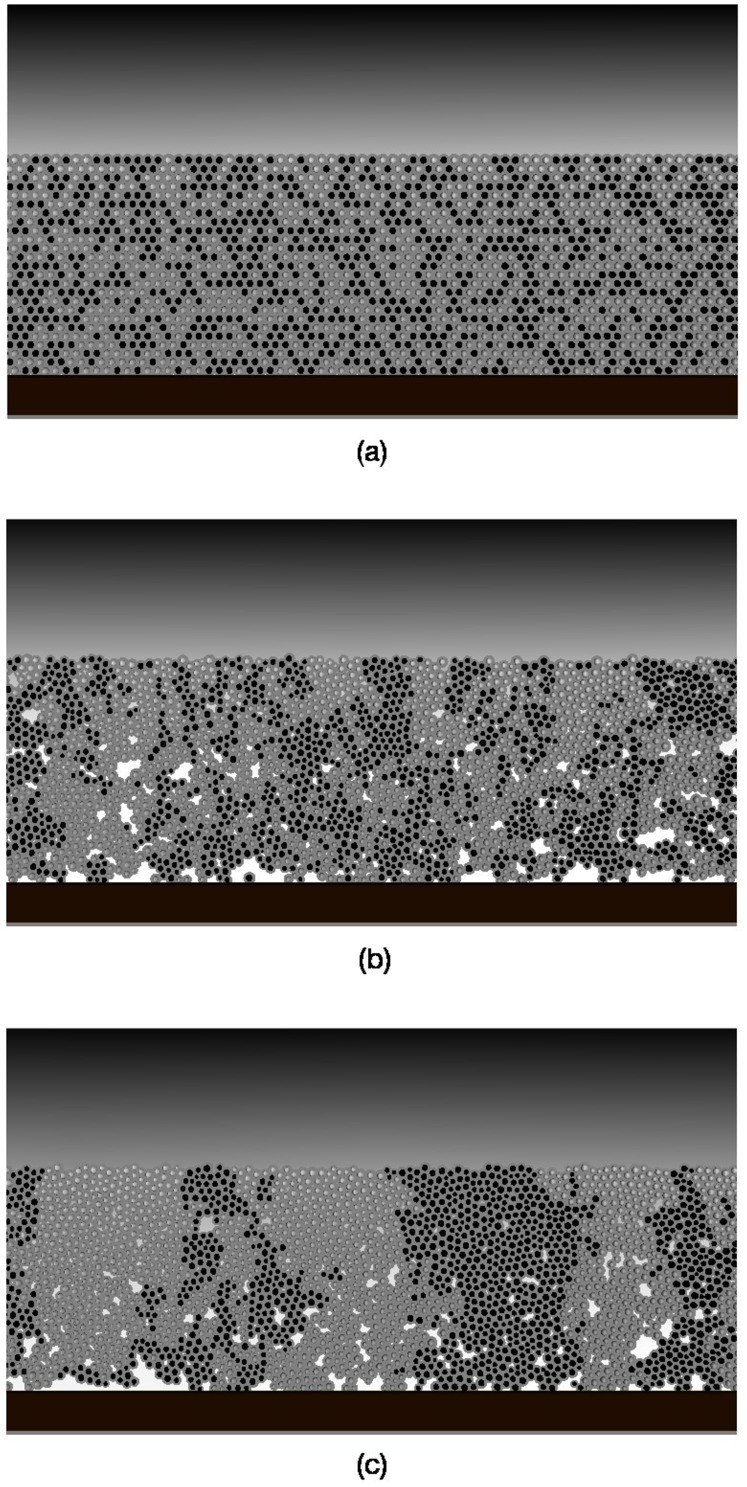
Snapshots of a section of a biofilm taken at time points (a) 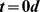, (b) 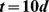 and (c) 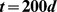 for the primary parameters in Table 1 (the full system size is roughly 10 times wider). Light grey (black) discs denote aggregates of A (NA), respectively, encased in EPS shells (grey). The shaded field in the background corresponds to the acid, with light (dark) regions for high (low) concentrations. The white regions near the base correspond to high concentrations of acid, visible in the voids created by cell death (see text). Larger, colour images and movies for 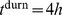 and 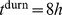 are available in Figure S1 and Movies S1 and S2 of the Supplementary Information.

Snapshots of a section of a biofilm for the primary parameters in Table 1 are given in Fig. 1 for the initial conditions at 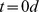, at an early time 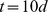, and at the final time point 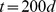. This time scale is relevant to biofilm accumulation at stagnant sites, such as the approximal surfaces between teeth. Even for the early time point there is visible aggregation of each distinct population compared to the initial condition, and these aggregates evolve into the depth-spanning domains visible at the later time. This is primarily a consequence of daughter particles remaining localised to their mother during division, driving the aggregation through shared proximity of descendants from the same progenitor. Note also that there is a clear gradient in the concentration of acid produced by the biofilm, with higher concentrations near the enamel surface and lower concentrations as one moves through the biofilm into the saliva layer, as seen in real plaque [37]. Lateral gradients in acid are not visible in these snapshots but are also present, in particular at late times when the large domains of A produce more acid than NA. Larger, colour images and movies are available in Figure S1 and Movies S1 and S2 of the Supplementary Information.

**Table 1:** Physical and biological parameters that were systematically varied in this study.

Label	Meaning	Primary value	Range
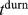	Duration of pulse cycle	6 h	2–10 h
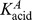	Half concentration for acid inhibition for A	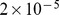 mol/L	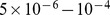 mol/L
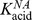	Half concentration for acid inhibition for NA	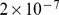 mol/L	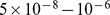 mol/L
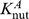	Half concentration for nutrient uptake for A	5 g/L	0.1–20 g/L
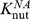	Half concentration for nutrient uptake for NA	20 mg/L	5–40 mg/L
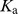	Effective dissociation constant	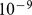 mol/L	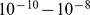 mol/L
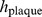	Thickness of the plaque layer	150 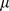 m	50–250 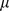 m
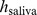	Thickness of the saliva layer	100 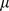 m	25–350 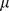 m
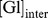	Concentration of sugar between pulses	5 mg/L	1–15 mg/L
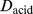	Diffusion coefficient for the acid (uniform)	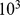 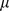 m^2^/s	140–2700 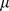 m^2^/s
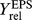	Relative yield factor for EPS	0.4	0.2–1.5
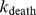	Linear factor in the kill rate	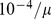 m h	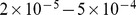 / 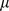 m h
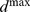	Threshold diameter for cell division	5 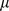 m	4 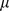 m–10 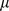 m
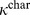	Characteristic stiffness	50 pN/ 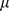 m	30–200 pN/ 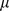 m
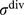	Width of daughter mass ratios after division	0.1	0.05–0.2

When one parameter was varied over the range given in the fourth column, the remaining parameters took the values given in the third column. See *Methods* for further details.

The two populations A and NA are not competing for the sole carbon source (glucose), since there is no mass transfer limitation of glucose as the model is defined. They are however competing for space, in that the fixed system size imposed by the plaque thickness 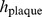 restricts the total number of particles of either population, 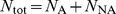, to remain roughly constant in time. Therefore, if one population exhibits a net growth rate faster than the other, it will increase its fraction within the biofilm at the expense of the other, whose fraction will contract. The only other form of direct interaction between the two populations is the acid produced which, particularly during the glucose pulse, severely reduces the growth of NA relative to A. Since A produces more acid that NA, this drives the formation of lateral pH gradients mentioned above.

### Alternating differential growth

The dominant population at late times is that with the greater net growth rate when averaged over both the inter and intra-pulse periods. If one population exhibited the greater rate in both periods, it would clearly outgrow the other. However, for this study we have chosen parameters for which population A exhibits the greater growth rate during the pulse when the pH is low, but population NA grows the fastest between glucose pulses when the pH is high. This situation is representative of oral bacteria as determined in chemostat experiments, for instance *S. mutans* is more aciduric that *S. gordonii*, but exhibits lower growth around neutral pH [38], [39]. Although the parameters were varied to cover a broad range of values, encompassing functionally similar species and strains, they were limited to ensure this basic alternation between the most competitive population remained true.

**Figure 2:**
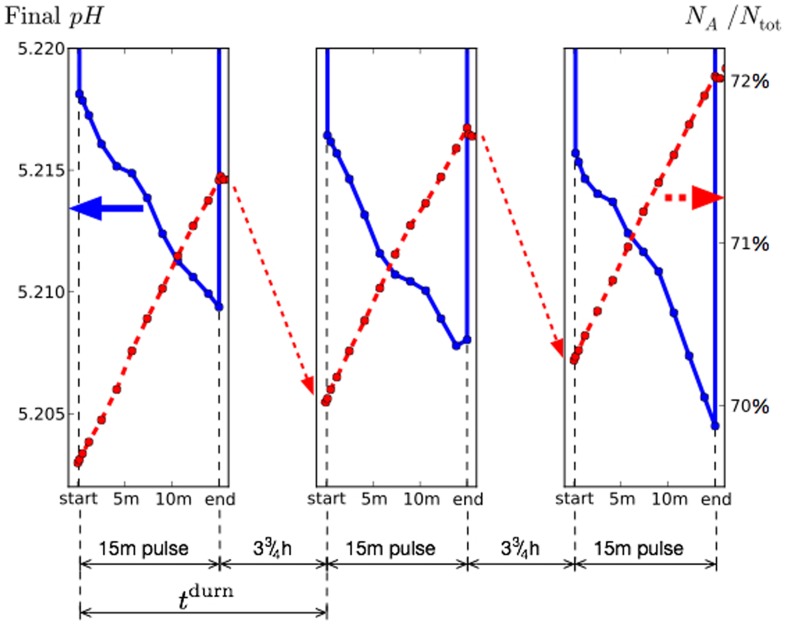
Example of evolution of the pH averaged over the tooth surface (left axis; solid lines) and fraction of biofilm that belongs to population A (right axis; broken lines) for 3 consecutive glucose pulses. This example is for the primary parameters of Table 1 except the total pulse cycle duration 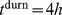 here. The pH is observed to decrease for successive pulses, concomitant with an increase in the fraction of A. During the inter-pulse period, which has been compressed for clarity, the fraction of A decreases slowly (shown schematically by the diagonal arrows), and the pH takes much higher values around 6.0 (not shown).

An example of this alternation is given in Fig. 2, which shows both the pH measured at the enamel surface, and the fraction of the biofilm that belongs to population A, *i.e.* 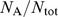, during three consecutive glucose pulses. For clarity of presentation the long inter-pulse periods have been compressed in this diagram, but the behaviour of both quantities can be inferred as explained in the caption. It is evident that the fraction 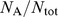 increases during the glucose pulse, but decreases again before the start of the subsequent pulse, when it again increases. Since A is more aciduric than NA, more acid should be produced as the fraction 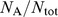 increases, and this is also evident in the figure where there is a clear correlation between pH and biofilm composition.

The example shown in Fig. 2 is for a somewhat frequent glucose pulsing with 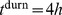. In this case, the increase in 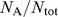 during the pulse is greater than the decrease between pulses. The long-term trend is therefore for A to dominate, and correspondingly the pH during pulses to drop. This can be regarded as a *ratchet* effect whereby each full pulse cycle increases the fraction of A by a small amount. The small shifts in composition and pH in the diagram lead to biologically significant changes when integrated over many such cycles. It might be expected that a longer 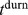, and hence a longer period of time spent in the high-pH environment, will result in a larger drop in 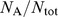 between pulses and a net decrease of 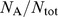, resulting in a slow drift towards a NA-dominated state. This is indeed observed, but instead we now consider data averaged over whole pulses for which ratcheting is not visible, to better focus on long-term trends.

### Transition between homeostatic and pathogenic biofilms

**Figure 3:**
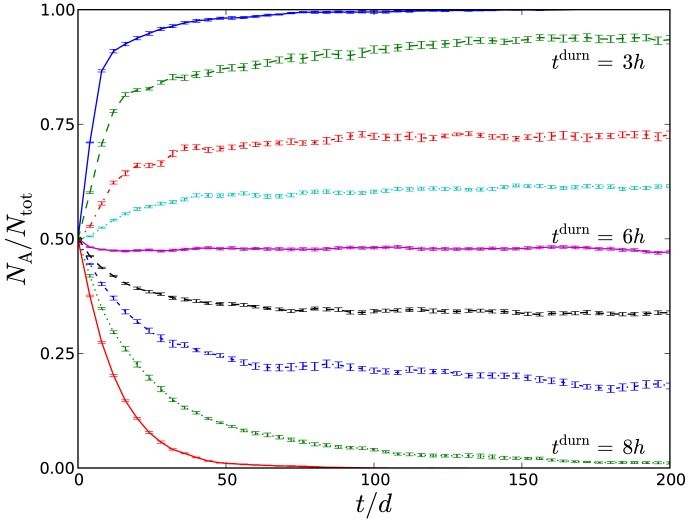
Fraction of system that belong to population A versus time for (from top to bottom) 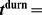 2 h, 3 h, 4 h, 5 h, 5.5 h, 6 h, 6.5 h, 7 h, 8 h, 10 h, respectively. Error bars show scatter over at least 10 independent runs.

Fig. 3 shows the fraction of population A versus time for pulse duration 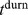 increasing from 2 h to 10 h, where each data point corresponds to the averaged value over a 2 d period so that the sawtooth variation in Fig. 2 is not visible. Starting from a 50∶50 population of A:NA, the biofilm composition becomes increasingly A-dominated with time for low 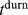 corresponding to frequent glucose pulses, *i.e.* frequent acid challenges. By contrast, for infrequent pulses with high 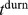, 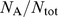 becomes small, signifying the biofilm becoming predominantly of type NA. Varying the frequency of glucose pulses in the biofilm environment therefore determines the late-time fate of the biofilm, *i.e.* whether it is A-dominated (pathogenic) or NA-dominated (homeostatic).

**Figure 4:**
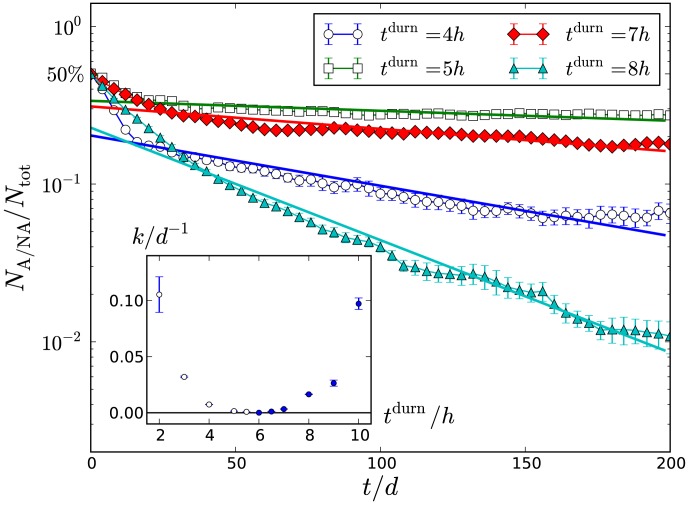
Plot of the fraction of the minority population (NA for open symbols, A for closed symbols) versus time, for the pulse cycle durations 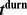 shown in the legend. Solid straight lines are the fit to exponential decay for times 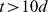. (Inset) Fitted rates versus 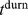.

It is evident from the figure that the biofilm composition 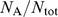 at any given time point continuously increases as the frequency of the glucose pulses increase. There is an intermediate value 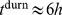 where the variation in biofilm composition is not discernible over the available data window. It can therefore be hypothesised that there is a transition value of 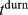 about which 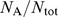 remains at 50∶50 for all times, and neither population comes to dominate the other. This is supported by quantitative analysis of the late-time variation of 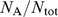, which can be shown to exponentially increase towards unity for low 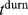, and exponentially decrease towards zero for high 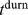. This is demonstrated by semi-logarithmic plot in Fig. 4, which shows examples of the exponential decay of A (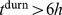) or NA (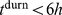). The fits in these figures are to a simple exponential,
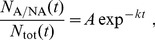
(1)where 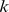 is the rate at which the minority fraction decays. The inset to the figure shows that this rate decreases continuously to zero as 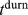 approaches a critical point close to 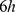. The exponential fit (1) is only successful if an initial transient of roughly 10 days is removed prior to fitting; no simple fit, including logistic growth, was found to fit the entire data range.

### Multi-factorial modulation of pathogenicity

The duration of glucose pulses 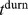 is not the only factor that determines the relative competitiveness of the two populations. Any of the model parameters listed in Tables 1 and 2 can, in principle, affect the growth rates of one or both populations over the course of a complete pulse, and therefore influence the selection of the dominant population. This is expected for the metabolic parameters such as the half-concentrations, but equally holds true for environmental factors. For example, increasing the biofilm thickness 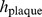 with all other parameters held fixed, results in an increased production of acid and a lower pH, reducing the metabolic rate of population A to a lesser degree than NA. This shift in competitiveness might be enough to make A the dominant population at late times, even when 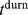 is greater than 6h.

**Table 2:** Physical and biological parameters that were not varied in this study and kept at the values shown.

Symbol	Description	Value	Reference
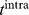	Duration of carbohydrate pulse	15 m	[41]
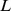	Longitudinal film width	2 mm	-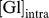	Concentration of sugar during a pulse	50 g/L	[38], [39], [47]–[50]
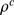	Cell density (excluding water)	0.2 pg/ 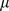 m^3^	[31]
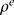	EPS density (excluding water)	4 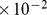 pg/ 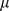 m^3^	[30]
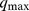	Base reaction rate	5/h	[38], [39]
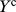	Yield factor for cell mass	0.1	[38], [39]
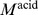	Molecular weight of (lactic) acid	90.08 g/mol	-

**Figure 5:**
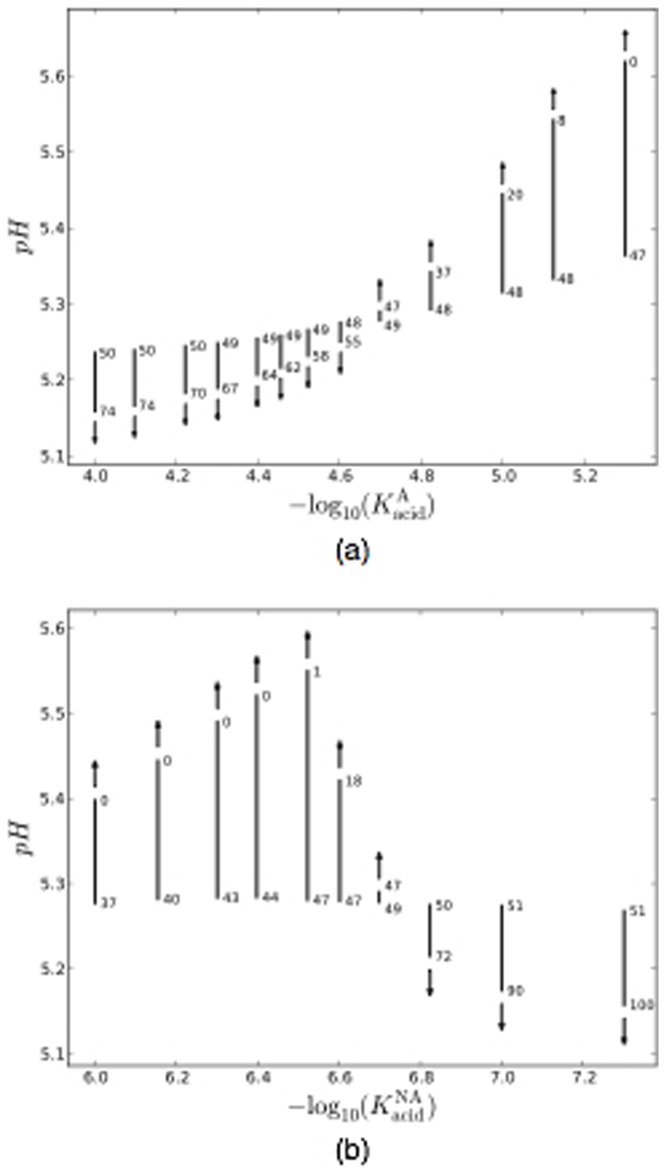
Variation of biofilm composition and pH during the glucose pulse as a single parameter is varied, for (a) 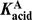 and (b) 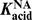. These parameters are plotted as 
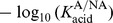
, corresponding to the pH at which cell metabolism is 50% inhibited by acidity. Each line segment shows the variation in pH, starting from 2 d and finishing on 200 d, with the arrow showing the direction of increasing time. The numbers show the percentage of biofilm occupied by A (*i.e.* 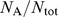) at the start and end points.

Confirmation that parameters other than 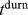 can promote a transition between pathogenic and homeostatic biofilms is presented in Fig. 5. This shows the variation of both the intra-pulse pH and the biofilm composition as a function of time, for a series of independent runs that differ only in one of the aciduricity parameters, 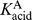 and 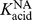. There is a clear trend from a late-time biofilm composition that is A-dominated with a low pH, arising for high 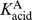 or low 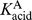, to a NA-dominated state with a higher pH for low 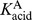 or high 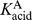. Thus, increasing the aciduricity of one population enhances its competitiveness with respect to the other. Note that in Fig. 5(b) the pH at the final time point first increases with the parameter 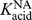 as it is increased, but then decreases with 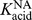 for the higher values considered. The initial increase is because of the increasing dominance of the NA population as just discussed. The subsequent decrease arises because, although the NA remain dominant, they become increasingly aciduric as 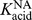 is increased, resulting in increased acid production. Indeed, increasing 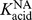 until it equals 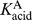 would result it two populations of equal aciduricity, rendering our labels A and NA meaningless.

**Figure 6:**
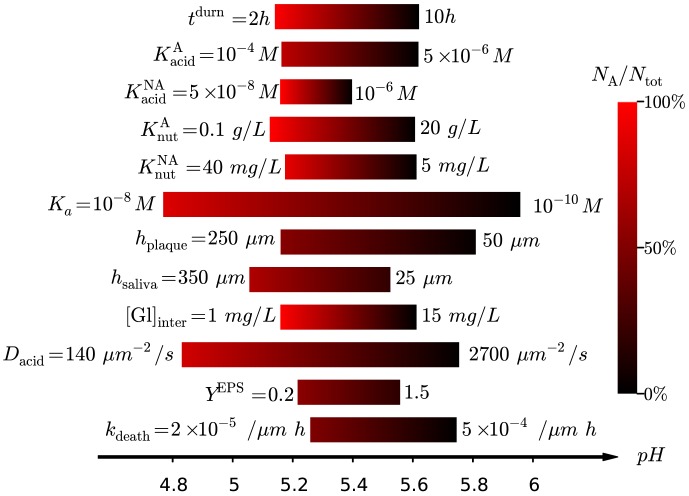
Variation of pH during the pulse at 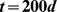 as the first 12 parameters of Table 1 are varied. Each bar corresponds to a single parameter being varied with the remaining held fixed at their primary values. The shading corresponds to the fraction of the biofilm occupied by population A as in the calibration bar.

Systematically varying each of the parameters in Table 1 reveals that most of them can also promote this transition. This is summarised in Fig. 6, which shows the biofilm composition and pH for 10 of the parameters between the extremes of the ranges given in the table. In all cases the observed variation is intuitive. For instance, increasing the diffusion 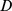 of acid results in more rapid dispersal from the system according to the boundary conditions of *Methods*, resulting in a higher pH and a lower fraction of A. Conversely, reducing the death rate 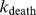 increases acid production, but this effect is weak as demonstrated below. The parameters 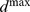, 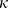 and 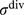 have been omitted from this figure since the signal-to-noise ratio was too small to discern any trend.

### Parameter sensitivity

**Figure 7:**
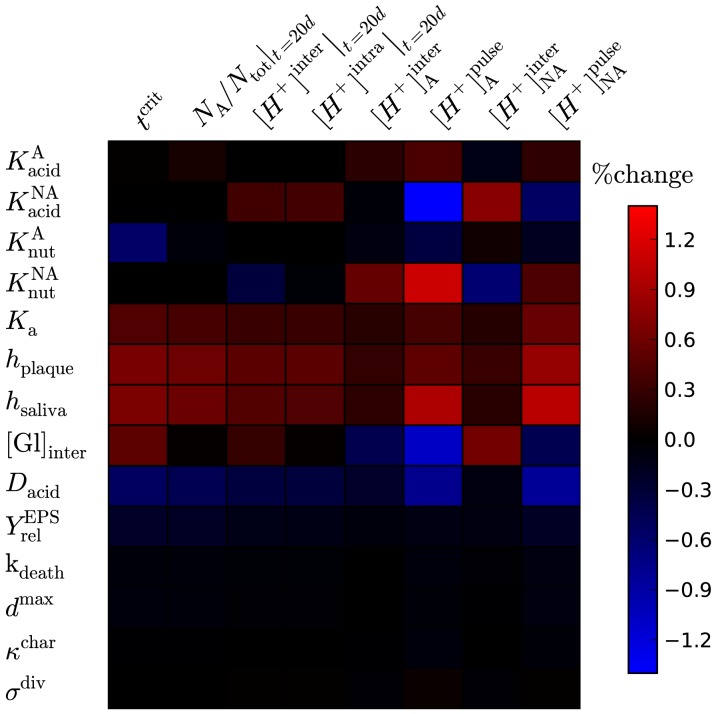
(Colour online) Sensitivity heat map between input parameters (rows) and measurable outputs (columns). Brightness corresponds to the relative change (increase or decrease) in the output quantity as the parameter is increased by 1%, as indicated by the colour bar. Actual values (with error bars) provided in Table S1 of the Supplementary Information.

It is not readily apparent from Fig. 6 which parameters are the most important for determining the late-time biofilm composition, making it unsuitable for identifying potential targets for controlling biofilm fate. In addition, the time point for the predicted quantities (pH and composition at 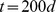) are not suitable for *in vitro* validation, for which a much shorter time scale is convenient. Both of these issues can be resolved by tabulating the changes in a range of putative experimental measurements with respect to each input parameter, allowing pairings that exhibit high or low sensitivity to be immediately identified. The sensitivity heat map for this model is Fig. 7, where each entry shows the predicted percentage change in a range of measurable outcomes for a 1% change in each input parameter. The measurable outcomes include 4 predictions for single-species biofilms, *i.e.* the concentration of 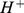 ions during and between pulses for systems comprised purely of A or NA, which reach dynamical steady states in the order of days. In addition, 4 outcomes for mixed-species films were considered. This includes the variation in the critical pulse duration 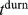 separating pathogenic from homeostatic biofilms, and 3 quantities measured at a time point of 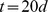 starting from an initial 50∶50 composition of A:NA, namely the fraction of 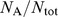, and the concentration of 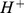 (again during and between pulses).

Inspection of the table immediately reveals that the quantity 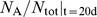 is the most sensitive to a number of parameters, suggesting this would be a useful quantity to measure in experiments. Single-species experiments may be the best to measure each species' metabolic half-constants, although environmental factors are clearly also important. Reading across rows rather than down columns suggests that the final 5 parameters in the diagram give weak or no change to the measurable outcomes, suggesting these mechanisms are not worthy of further investigation. The variations are not necessarily zero, as seen by inspection of the raw data given in Table S1 of the Supplementary Information, but their influence is evidently weak.

## Discussion

Mathematical modelling represents a powerful tool for biofilm investigation, providing the capability for quantitative prediction upstream from *in vitro* models and *in vivo* trials. All conceivable measures of biomass composition, structure, and associated chemical gradients can be extracted non-invasively from an *in silico* biofilm. In addition, such data are acquired in an accelerated time frame, which for the results presented here translates into 200 days real time in approximately 10 hours of simulation time, and this ratio could be further improved with additional numerical optimisation and parallelisation. This rapidity allows ranges of each input parameter to be systematically assayed and the corresponding effect on the growing biofilm to be determined, both qualitatively in terms of the nature of the changes, and quantitatively in terms of if these changes are significant or slight.

The freedom with which parameters can be varied makes this form of modelling well suited to studying oral biofilms. The microbial composition of these complex multi-species communities varies greatly between human hosts, with phylogenetically distant organisms fulfilling overlapping functional roles. Focussing on microbial function rather than genetic identity is therefore desirable when developing clinical treatments, and mathematical modelling facilitates this by permitting the rapid assaying of parameters that vary between functionally-similar species. This should be contrasted with the equivalent *in vitro* experiments, which would need many repeats with different species, strains and sub-strains to sample equivalent ranges of metabolic activity.

When applied to competing acidogenic populations varying in their rates of nutrient uptake 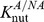 and aciduricity 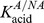 as in this work, this approach demonstrates that these parameters measurably affect the cariogenic potential of (supragingival) oral biofims; see Fig. 7. This insight can be used to suggest treatments for modulating biofilm composition towards a benign homeostatic state, and indeed reducing aciduricity is one mechanism by which fluoride promotes improved oral health [12], [13]. This highlights the potentially controlling role of sublethal treatments in modulating population dynamics. It also highlights an additional advantage of mathematical modelling, in that it allows us to isolate this one mechanism from many other postulated roles for fluoride, simply because these alternatives were not included in the model.

Confirmation of the predictive capability of this or any model requires model validation. The parameter sensitivity map in Fig. 7 can serve as a framework for model validation, in two respects. Firstly, it helps identify experiments that can be used to estimate specific model parameters. If the sensitivity of any single experimental measure with respect to a given parameter is high, corresponding to bright entries in the table, the resulting estimate will have a low signal-to-noise ratio and thus should be reliable. Secondly, parameters for which the sensitivity is low need not be determined to any degree of precision for reliable model predictions. It is clear from Fig. 7 that the parameters for cell division, stiffness and death rates have little effect on any of the postulated experimental measures, suggesting that rough approximation of their values should suffice.

Parameter sensitivity can also be used to suggest directions for further model development. It is apparent from Fig. 7 that the parameters for inter-pulse glucose concentration 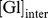 and acid buffering 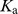 have a measurable influence on the resulting predictions. Both of these mechanisms were incorporated in an approximate manner in this work, and this sensitivity suggests that further modelling would benefit from expanding each mechanism to include more detail, albeit with the overhead of an increased number of parameters. Indeed, series of reactions for both glucose storage and acid buffering have already been specified for the non-growing biofilms of Ilie *et al.* [27], and these could be incorporated in an agent-based model such as ours.

The real plaque ecosystem maintains a dynamic equilibrium between multiple species whose relative fractions vary with environmental conditions, but never vanish entirely [1]. By contrast, here one species type is progressively removed from the system, as long as the frequency of glucose intake and low-pH challenges remains constant. This most likely represents the simplicity of the model, and many model extensions are likely to permit subpopulations of functionally dissimilar species to be perpetually maintained. Additional community complexity in the form of more than two distinct species types interacting *via* a range of interactions, mutualistic, antagonistic and otherwise, should permit a dynamically stable biofilm composition. Heterogenous biofilm composition leads to chemical gradients and an extended habitat range for species that would not be able to persist in a homogenous biofilm, features that can be included in agent based modelling. Other possibilities for maintaining a dynamic equilibrium have been discussed elsewhere [6]–[8]. Finally, we notice that clonal variation was not included in this version of the model, so all members of a population are phenotypically identical. It is, however, straightforward to introduce such a feature into agent-based modelling, at the expense of additional parameter fitting.

## Analysis

The mathematical model employed here is based on the Individual based Model (IbM), an established agent-based model for biofilms that has been applied to a range of bacterial communities and environments [28]–[36]. Such models consist of two coupled phases, a particulate phase where each particle represents a bacterium or bacterial aggregate, and a series of overlapping continuous phases representing the concentration fields of one or more dissolved species, *e.g.* nutrients or metabolic products. The variant here admits biomechanics in that the particles are interconnected *via* springs representing adhesion by the extracellular polymeric substances (EPS). Here we summarise only those features of the model relevant to the subsequent discussion, and direct the reader to [40] for further details.

### Model overview

**Figure 8:**
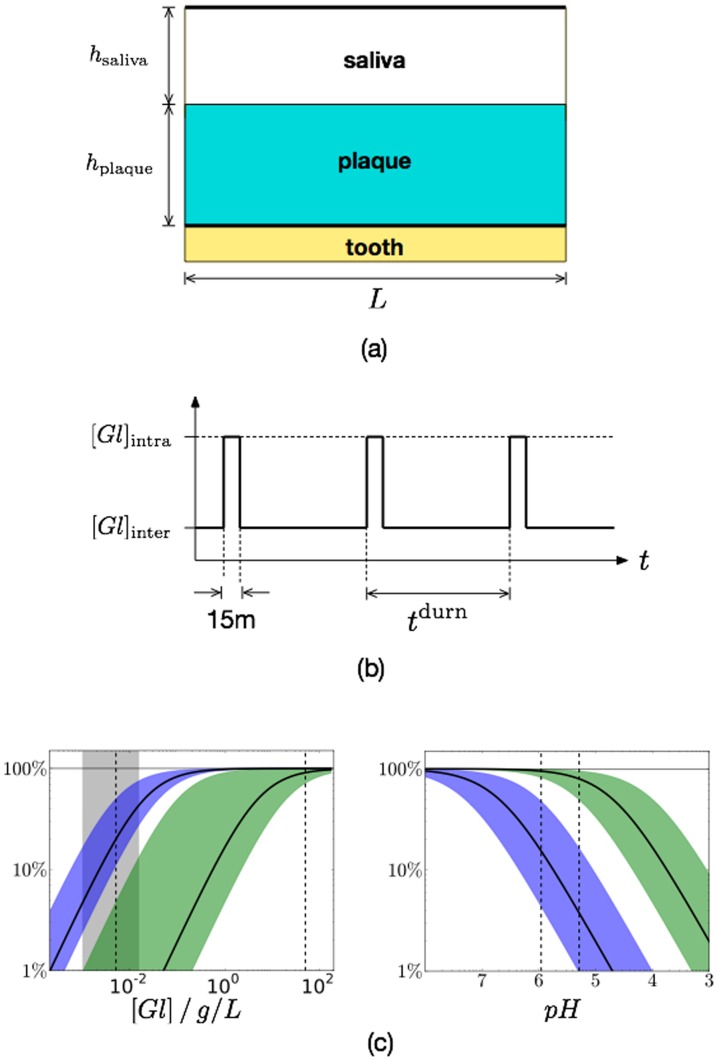
Schematic of the model. (a) The system geometry. (b) The temporal variation of the environmental glucose follows a feast-famine protocol with a total cycle time 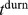, including the 15 minute pulse. (c) Relative metabolic rates due to nutrient (left) and acid inhibition (right) as per (2), so 100% means saturation and 1% means a significant reduction. For both plots, the leftmost solid curve corresponds to the primary value for NA, and the right to A. Each curve also has a shaded region giving the total range considered for each parameter. For the left plot, the parameters for 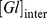 and 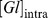 are shown as vertical dashed lines, with the shaded region for the former showing the range considered. For the right plot, the vertical dashed curves show typical pH values during (right) and in between (left) glucose pulses for the primary values in Table 1.

In this study we consider a mixed film consisting of two microbial populations that differ in their ability to metabolise sugars in the presence of low pH. These are referred to as A for aciduric and NA for non-aciduric, and parameters relating to each species are labelled A or NA accordingly. Additionally there is a single scalar field representing the concentration of lactic acid produced by each particle's glycolysis of dietary sugar. The system domain is schematically shown in Fig. 8(a). A two-dimensional geometry has been employed as this permits biofilms of lateral extent far exceeding their thickness to be simulated within a reasonable timeframe. Periodic boundaries have been assumed in the direction parallel to the enamel surface.

The nutrient (glucose) is not represented as a spatially-varying field, but is instead assumed to have a uniform concentration with no gradients. The concentration does however vary in time as shown in Fig. 8(b). This follows a feast-famine protocol representing the dietary intake of fermentable carbohydrates, whereby the concentration 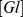 alternates between short periods at a high value 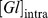, interspersed with extended periods at a lower value 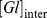. 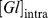 is taken to be far above the half-concentrations for nutrient uptake (see below), so the metabolism for both populations is saturated during the pulse. The duration of the pulse has been fixed at 15 minutes as this represents a typical removal time of acid from the oral cavity [1], [41]. The duration of a complete pulse cycle (*i.e.* inter plus intra periods), 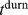, is a key parameter of the model.

The model parameters are listed in two separate tables, where the free parameters that were systematically varied in this study are listed in Table 1, and the fixed parameters whose values were estimated from the literature and not varied are given in Table 2. Additional numerical parameters, such as the convergence parameters for the chemical and mechanical relaxation, were tested to be sufficiently small to not affect the results and are not quoted here.

### Cell metabolism

Acid buffering within plaque results in a lower concentration of 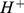 ions, and therefore a higher pH, than for the same concentration of lactic acid in aqueous solution [41]. However, empirical titration curves cannot be easily incorporated into models as discussed elsewhere [23]. Rather than include a series of coupled reactions as in [27], which would slow down our simulations and introduce additional parameters, we instead treat the dissociation of lactic acid to 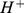 as a single-step process with an *effective* dissociation constant 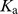 that is far lower in value than aqueous dissociation. 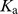 thus becomes a free parameter which we systematically vary. The concentration of glucose between pulses, which depends on the balance of storage and conversion reactions, is also simplified to a single free parameter 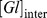.

The glycolysis of glucose to lactic acid is assumed to depend on two factors, the concentration of glucose 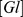 and the local acidity 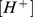. Both quantities modulate the overall reaction rate as independent Monod factors with half-concentrations 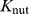 and 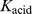 for nutrient uptake and acid inhibition respectively, as shown in Fig. 8(c). The full expression for the reaction rate 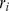 (mass per unit time) for the particle with label 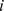 and mass 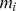 is
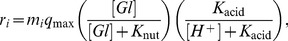
(2)where 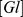 is in units of mass per unit volume, and 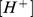 in molarity. Each half-concentration has the same units as its corresponding concentration, and have separate values for A and NA, *e.g.* 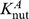 and 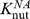.

The spatial distribution of lactic acid, which is both produced by (2) and modulates it by determining 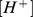, obeys the standard reaction-diffusion equation in which local production of acid is balanced by diffusion away from the source. The primary value for the diffusion coefficient 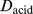 is taken to be that for lactic acid in water, but was also systematically varied. The reaction-diffusion equation was numerically solved using geometric multi-grid on a rectangular mesh [40], with no-flux boundary conditions at the enamel surface and the requirement that the acid at the upper surface of the saliva layer is zero.

Once the 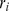 for each particle 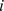 is determined, the resulting rate of increase in mass is computed as 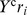, where 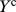 is the dimensionless yield factor; here we fix 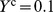 for both bacterial populations, comparable to representative oral bacteria [38], [39]. EPS is assumed to be produced at a rate proportional to the cell mass, *i.e.* the mass of the EPS increases at a rate 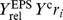, where 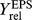 is a free parameter that we systematically vary. Particle and EPS masses are converted to physical size by assuming fixed densities of both components as listed in Table 2.

When a particle diameter exceeds the critical value 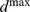 it divides into two daughter particles. The mass of the mother particle, 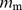, is conserved but is distributed asymmetrically to the daughters, *i.e.* with masses 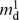 and 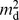 obeying
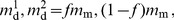
(3)where 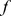 is a random number drawn from a Normal distribution with mean 0.5 and width 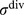. The mass of the EPS is distributed similarly, with the same 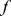. The two parameters 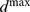 and 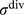 are treated as free and systematically varied.

### Redistribution and removal of biomass

After particle growth and division, the particles are rearranged so as to ensure the biofilm as a whole is mechanically stable. This procedure, described in detail in [40], involves constructing a network of springs connecting nearby particles. Each of these springs has a stiffness that is proportional to the local EPS mass and the model parameter 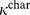. In addition, springs between particles and the enamel surface have a stiffness that is also proportional to 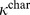. The free parameter 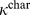 is here varied around values suggested by AFM experiments [42] as shown in Table 1. The numerical procedure involves converting the requirement of mechanical equilibrium to a sparse matrix equation and solving using the conjugate gradient method. This matrix approach has also been adopted for plant biofilms, motivated by numerical performance [43].

The plaque biofilm is limited in thickness to 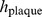, in that any particles whose centre exceeds this height is removed from the system. This can be thought of as a simplistic representation of biomass removal due to fluid shear by flowing saliva [32], and can be achieved *in vitro* by a constant depth film fermenter [13], [44]–[46]. Above the plaque biofilm is the saliva layer of thickness 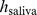 in which there is no biomass, but the concentration of lactic acid still continuously varies until vanishing at the upper saliva surface.

Additionally, since bacteria deep within a biofilm exhibit a lower viability than those near the surface, we include a second mechanism for particle removal, namely that cells are ‘killed’ at a rate that depends on their distance from the exposed biofilm surface, with the highest death rate near the enamel. The death rate per unit depth per unit time is denoted 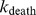 and is a free parameter that is varied here. This parameter is the same for both populations.

### Parameter sampling

Given the large number of parameters, a systematic investigation of all combinations was not feasible. Instead, each free parameter was assigned a primary value based on chemostat experiments of oral bacteria [38], [39], known environmental conditions for oral biofilms [1], [7], [8] and related models [27]. The primary value for the effective dissociation constant 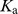 was selected to give a realistic pH during the glucose pulse, and that for 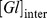 to give a critical value of 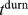 (see below) around 6 h. Each parameter was then varied over the range specified in Table 1 with all other parameters held fixed.

The sensitivity heat map discussed in *Results* refers to linear response, *i.e.* small changes in the input parameters for which the corresponding change in each output parameter was proportional. For each model parameter, a range of variations was tested to ensure the corresponding variation in the outcome was linear (unlike the data in Fig. 6, which is concerned with non-linear trends over a broad variation of parameters), which was typically the case for a 5–10% change in the model parameter. The sensitivity was then scaled to a 1% value. The raw data (with errors) are given in Table S1 of the Supplementary Information.

## Supporting Information

Figure S1
**Snapshots of examples biofilms.** (a) 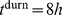 at a time point of 10 days, (b) the same after 200 days, and (c) 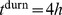 after 200 days. Green (blue) discs correspond to populations of A (NA), respectively. The red in the background corresponds to the lactic acid, with red (black) corresponding to high (low) concentrations respectively. All other parameters are the same as Fig. 1 in the main article.(PDF)

Table S1 **Raw data for the sensitivity heap map.** Values correspond to the percentage change in the measurable outcome (columns) for a 1% change in the model parameter (rows). Error bars are either given explicitly, or as variation in the final 2 digits (shown in brackets).
(PDF)

Movie S1
**Movie corresponding to Fig. S1(b).** Extends from 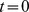 to 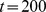 days. Colour coding and parameters as per Fig. S1(b).(MP4)

Movie S2
**Movie corresponding to Fig. S1(c).** Extends from 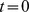 to 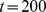 days. Colour coding and parameters as per Fig. S1(c).(MP4)
