# Supplementary material for: Non-Lethal Control of the Cariogenic Potential of an Agent-Based Model for Dental Plaque
Source: PLoS One. 2014 Aug 21;9(8):e105012. doi: 10.1371/journal.pone.0105012 (PMC4140729; doi:10.1371/journal.pone.0105012)
Supplement: Figure S1 — Snapshots of examples biofilms. (a) at a time point of 10 days, (b) the same after 200 days, and (c) after 200 days. Green (blue) discs correspond to populations of A (NA), respectively. The red in the background corresponds to the lactic acid, with red (black) corresponding to high (low) concentrations respectively. All other parameters are the same as Fig. 1 in the main article. (PDF) [file pone.0105012.s001.pdf]

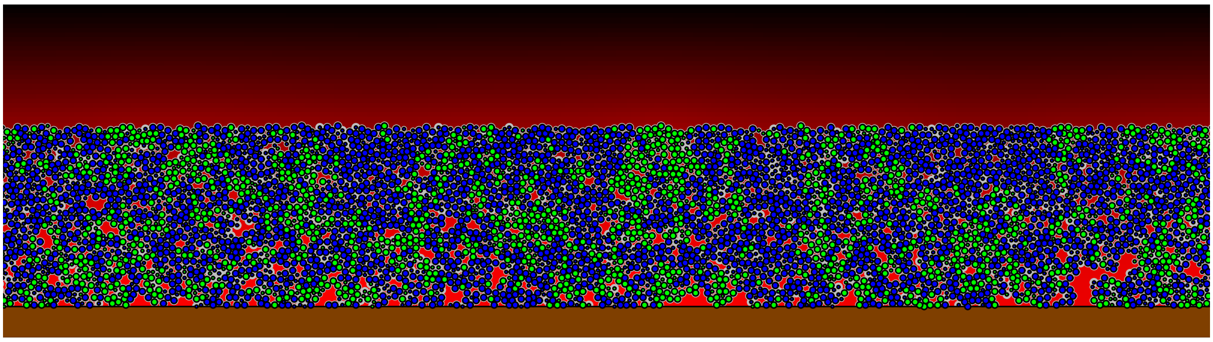

(a)

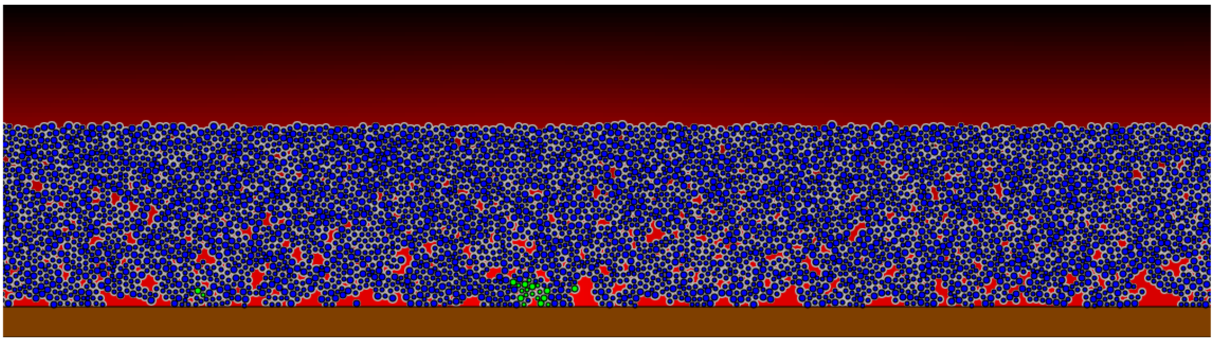

(b)

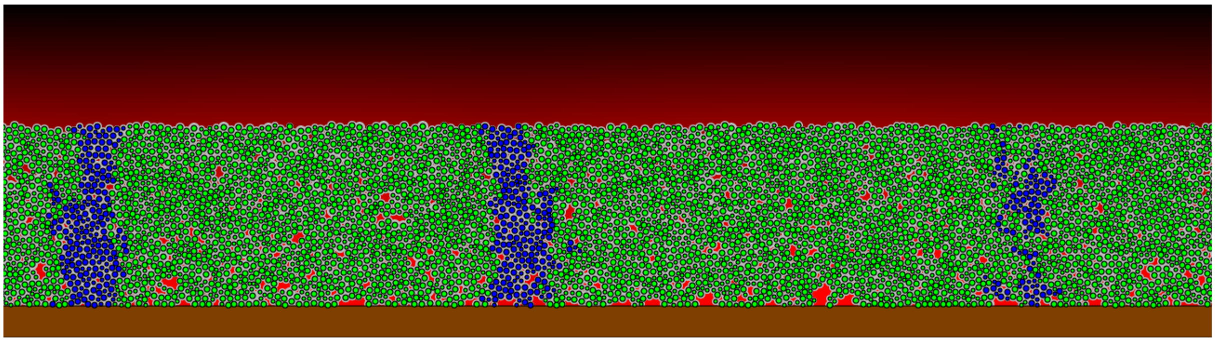

(c)

Figure S1: **Snapshots of examples biofilms.** (a) $t^{\mathrm{durn}} = 8h$ at a time point of 10 days, (b) the same after 200 days, and (c) $t^{\mathrm{durn}} = 4h$ after 200 days. Green (blue) discs correspond to populations of A (NA), respectively. The red in the background corresponds to the lactic acid, with red (black) corresponding to high (low) concentrations respectively. All other parameters are the same as Fig. 1 in the main article.
