# Supplementary material for: Non-Lethal Control of the Cariogenic Potential of an Agent-Based Model for Dental Plaque
Source: PLoS One. 2014 Aug 21;9(8):e105012. doi: 10.1371/journal.pone.0105012 (PMC4140729; doi:10.1371/journal.pone.0105012)
Supplement: Table S1 — Raw data for the sensitivity heap map. Values correspond to the percentage change in the measurable outcome (columns) for a 1% change in the model parameter (rows). Error bars are either given explicitly, or as variation in the final 2 digits (shown in brackets). (PDF) [file pone.0105012.s002.pdf]

Parameter	$[H^+]_{\rm A}^{\rm inter}$	$[H^+]_{\rm A}^{\rm pulse}$	$[H^+]_{\rm NA}^{\rm inter}$	$[H^+]_{\rm NA}^{\rm pulse}$	$t^{\rm crit}$	$N_{\rm A}/N_{\rm tot}\|_{t=20d}$	$[H^+]^{\rm inter}\|_{t=20d}$	$[H^+]^{\rm intra}\|_{t=20d}$
$K_{\rm acid}^{\rm A}$	0.019±0.025	0.125(23)	0	0	0.239(60)	0.40(17)	-0.121(54)	0.254(75)
$K_{\rm acid}^{\rm NA}$	0	0	0.352(19)	0.370(18)	-0.050±0.060	-1.40(27)	0.749(85)	-0.54(12)
$K_{\rm nut}^{\rm A}$	-0.559(12)	-0.063(10)	0	0	-0.078(28)	-0.35(10)	0.096(33)	-0.181(45)
$K_{\rm nut}^{\rm NA}$	0	0	-0.3283(97)	-0.0340(90)	0.556(36)	1.11(11)	-0.615(31)	0.419(43)
$K_{\rm a}$	0.4429(36)	0.3904(32)	0.3164(49)	0.3122(47)	0.209(15)	0.373(42)	0.202(14)	0.564(18)
$h_{\rm plaque}$	0.658(66)	0.600(61)	0.495(51)	0.500(48)	0.280(73)	0.52±0.31	0.316(91)	0.81(12)
$h_{\rm saliva}$	0.672(63)	0.589(58)	0.452(44)	0.427(45)	0.25±0.13	0.95±0.55	0.22±0.16	1.00(22)
$[{\rm Gl}]_{\rm inter}$	0.512(11)	0.024(10)	0.292(10)	0.0278(93)	-0.436(31)	-1.08(10)	0.627(32)	-0.445(43)
$D_{\rm acid}$	-0.521(40)	-0.451(35)	-0.344(46)	-0.332(45)	-0.231(96)	-0.77(38)	-0.08±0.11	-0.83(14)
$Y_{\rm rel}^{\rm EPS}$	-0.228(27)	-0.201(24)	-0.122(16)	-0.116(15)	-0.068(27)	-0.10±0.12	-0.097(38)	-0.198(54)
${\rm k_{kill}}$	-0.063(11)	-0.057(11)	-0.0383(91)	-0.0336(86)	0.005±0.029	-0.07±0.10	-0.031±0.031	-0.078±0.040
$d^{\rm max}$	-0.072(15)	-0.062(15)	-0.028(10)	-0.0282(85)	0.008±0.028	-0.05±0.15	-0.014±0.044	-0.077±0.059
$\kappa^{\rm char}$	-0.0205(78)	-0.0174(71)	-0.009±0.004	-0.008±0.004	-0.016±0.028	-0.07±0.11	0.010±0.033	-0.047±0.046
$\sigma^{\rm div}$	0.0±0.013	0.0±0.012	0.016±0.009	0.016±0.009	-0.022±0.027	0.06±0.15	-0.024±0.046	0.019±0.061

Table S1: **Raw data for the sensitivity heap map.** Values correspond to the percentage change in the measurable outcome (columns) for a 1% change in the model parameter (rows). Error bars are either given explicitly, or as variation in the final 2 digits (shown in brackets).
